# Portal Vein Embolization with PVA and Coils before Major Hepatectomy: Single-Center Retrospective Analysis in Sixty-Four Patients

**DOI:** 10.1155/2019/4634309

**Published:** 2019-10-10

**Authors:** R. Camelo, J. H. Luz, F. V. Gomes, E. Coimbra, N. V. Costa, T. Bilhim

**Affiliations:** ^1^Radiology Department, Hospital de São José, CHLC, 1150-199 Lisbon, Portugal; ^2^Interventional Radiology Department, Centro Hepato-Bilio-Pancreático e de Transplantação, Hospital Curry Cabral, CHLC, 1069-166 Lisbon, Portugal; ^3^Nova Medical School, Faculdade de Ciências Médicas, Universidade Nova de Lisboa, Lisbon, Portugal; ^4^Head Interventional Radiology Department—Centro Hepato-Bilio-Pancreático e de Transplantação, Hospital Curry Cabral, CHLC, Lisbon, Portugal

## Abstract

**Objectives:**

Portal vein embolization (PVE) stimulates hypertrophy of the future liver remnant (FLR) and improves the safety of extended hepatectomy. This study evaluated the efficacy of PVE, performed with PVA and coils, in relation to its effect on FLR volume and ratio. Secondary endpoints were the assessment of PVE complications, accomplishment of liver surgery, and patient outcome after hepatectomy.

**Materials and Methods:**

All patients who underwent PVE before planned major hepatectomy between 2013 and 2017 were retrospectively analyzed, comprising a total of 64 patients. Baseline patient clinical characteristics, imaging records, liver volumetric changes, complications, and outcomes were analyzed.

**Results:**

There were 45 men and 19 women with a mean age of 64 years. Colorectal liver metastasis was the most frequent liver tumor. The majority of patients (*n* = 53) had a right PVE. FLR increased from a mean value of 484 ml ± 242 to 654 ml ± 287 (*p* < 0.001) after PVE. Two major complications were experienced after PVE: 1 case of left hepatic artery branch laceration and 1 case of hemoperitoneum and hemothorax. A total of 44 (69%) patients underwent liver surgery. Twenty-one patients were not taken to surgery due to disease progression (*n* = 18), liver insufficiency (*n* = 1), and insufficient FLR volume (*n* = 1), and one patient declined surgery (*n* = 1).

**Conclusions:**

PVE with PVA and coils was accomplished safely and promoted a high FLR hypertrophy yield, enabling most of our patients to be submitted to the potentially curative treatment of liver tumor resection.

## 1. Introduction

Liver resection of hepatic tumors is the firstline treatment option for curative intent in hepatic malignancies, and in order to accomplish free surgical margins, an extended hepatectomy is required up until 45% of liver tumors [1]. However, the main cause for not performing the planned hepatic resection is inadequate future liver remnant (FLR) volume before surgery. Consequently, FLR size must be optimized to prevent postoperative liver failure (PLF), the principal cause of postoperative death after major hepatectomy [2]. In order to extend the indications of main hepatic resection and to prevent PLF, preoperative portal vein embolization (PVE) has been performed through the last decades, allowing atrophy of the future resected liver segments and hypertrophy of the FLR [3, 4].

It is suggested an FLR to total functional liver volume (TFLV) ratio of at least 25% in patients without hepatic dysfunction, and minimum ratios of 35 to 40% in patients with compromised hepatic function (e.g., obstructive jaundice, chronic liver disease, or intensive chemotherapy) [5–10]; however, the minimum total hepatic volume required to avoid PLF has not been precisely determined. PVE has a high technical success rate approaching 100% in most of the series [11], and only a small number of unsuccessful techniques have been reported [12, 13]. The resection rate after PVE must be about 80 to 85%, although this rate may decrease to 70% in cirrhotic patients. The main reasons for not performing the liver resection after PVE are local tumor progression and peritoneal or other metastases discovered at the follow-up computed tomography (CT), magnetic resonance imaging (MRI), or laparotomy. Insufficient hypertrophy after PVE is rare, occurring in less than 10% of the patients in secondary liver malignancies; however, it can occur in up to 20% cirrhotic patients [11, 14].

PVE is considered safe and effective, and many hepatobiliary units worldwide adopt it as their principal strategy for FLR increase before major hepatic resection. Other approaches for preoperative hepatic augmentation have been used such as arterial embolization, hepatic vein embolization, and portal vein ligation. Once compared with arterial embolization, PVE presents lower toxicity not only because side effects are minor but also because signs and symptoms of postembolization syndrome (e.g., nausea and vomiting, fever, and pain) are uncommon. Abnormal liver function after PVE is frequently subtle and temporary, and about 50% of patients have no considerable change [2].

Since one of the most important properties of an embolic material is its capacity to induce FLR hypertrophy when used for PVE, we wanted to access this specific outcome in our own series of patients at our high-volume liver surgery and transplant center.

## 2. Materials and Methods

### 2.1. Patient Population

The Institutional Review Board of our center approved this study protocol. Between 2013 and 2017, all patients treated with PVE before planned major hepatectomy were identified. Baseline patient clinical characteristics, imaging records, liver volumetric data, and postoperative course were collected retrospectively.

### 2.2. Inclusion and Exclusion Criteria

**Figure 1:**
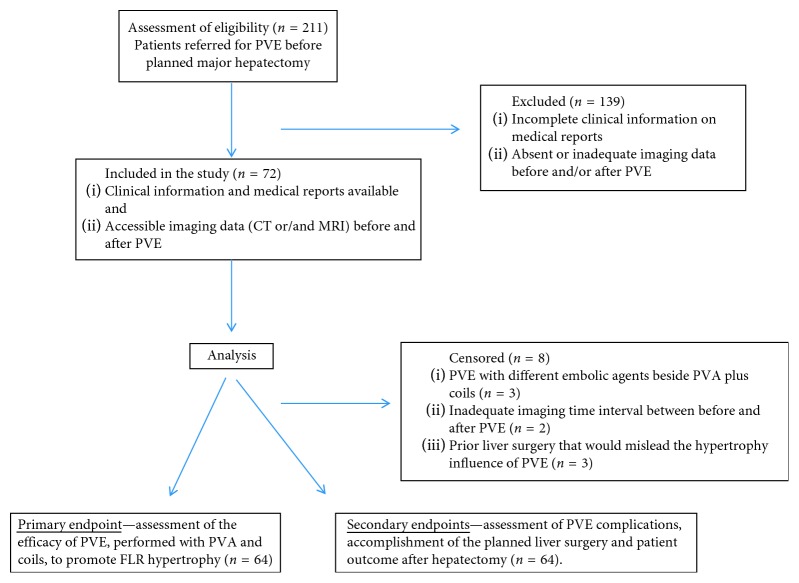
Patient flow chart.

All patients who underwent PVE before planned major hepatectomy between 2013 and 2017 were retrospectively analyzed. Exclusion criteria were as follows: unavailable or inadequate imaging data (CT and/or MR) before and after PVE, previous segmentectomy and/or hepatectomy, and PVE with other embolic agents beside PVA plus coils. The analyzed cohort comprised 64 patients (Figure 1).

### 2.3. Study Endpoints

Our main endpoint was to assess the efficacy of PVE, performed with PVA and coils, in relation to its effect on FLR volume and ratio. Secondary endpoints were the assessment of PVE complications, attainment of hepatic surgery, patient outcome after liver resection, and survival.

### 2.4. PVE Technical Considerations

Patients were allocated to a hospital bed, with an anticipated 24 h hospitalization, before the PVE procedure. The PVE technique adopted in our institution has been described elsewhere [13, 15]. In brief, the portal vein was accessed through a transhepatic ultrasound-guided puncture. The ipsilateral portal vein approach (the liver puncture is accomplished in the tumor bearing liver lobe and not the FLR) was adopted when possible, always avoiding tumor transgression. A branch from the anterior sectorial right portal vein was preferentially punctured instead of a branch from the posterior sector. A micropuncture kit (MAK—Merit Medical, South Jordan UT, USA) was used to access the portal vein. Portal angiography (Philips angiography suite FD-20, Netherlands) was performed, using a reversed curve catheter Simmons II 4F (Cordis, USA), to assess the anatomical pattern of the portal vein, through an automated injector with a 25 ml volume of contrast at a 7 ml per second flow protocol. Using the same 4F catheter, catheterization and embolization of non-FLR portal branches with PVA particles (Merit Medical) was performed first to achieve flow stasis. PVA particles from 150 to 700 *μ*m in size were injected in a stepwise fashion. Smaller particles (150 to 250 *μ*m) were infused primarily until significant decrease in forward flow was detected. This form of distal embolization is thought to constraint development of collateral circulation that may potentially limit hypertrophy [13]. Metallic pushable 0.035-inch coils (Cook Medical, Bloomington, IN, USA) were then deployed proximally to inhibit venous inflow and subsequently decrease the possibility of recanalization. Likewise, with PVA particles, smaller size coils are deployed more distally in the portal vein branches, such as 6 mm in diameter, and up to 12 mm diameter coils are deployed more proximally. A postembolization direct portography is acquired to ensure proper embolization of the aimed portal branches and to check for any immediate complication such as coil migration. Gelfoam slurry embolization of the percutaneous transhepatic tract to the portal vein branch was performed to finish the procedure. During the PVE procedure, intravenous prophylactic antibiotics were permanently administered, and hospital discharge patients were posteriorly kept on oral analgesic administration, as required.

### 2.5. Volumetric Assessment of Future Liver Remnant: Primary Outcome

**Figure 2:**
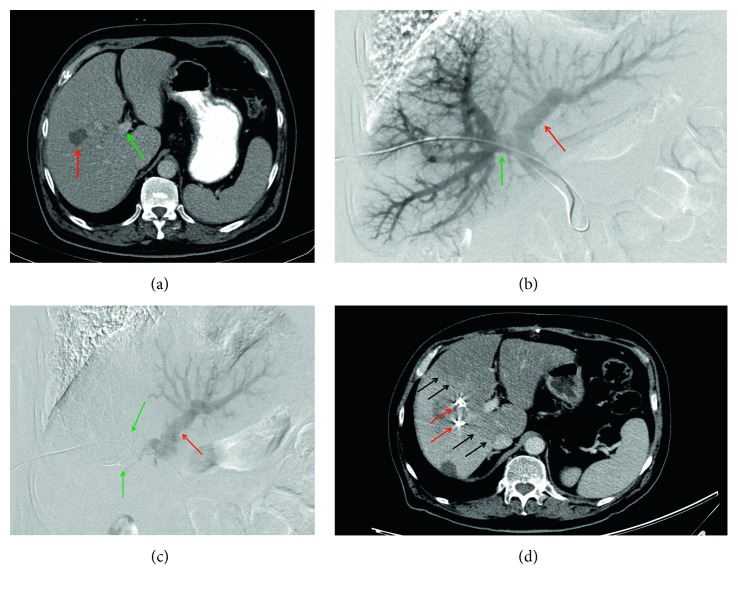
(a) A 66-year-old male with colorectal cancer presenting with right liver lobe metastasis. Computed tomography shows a small left liver (the planned surgery was a right hepatectomy), insufficient for the future right hepatectomy resection. Red arrow: liver metastasis. Green arrow: left portal vein. (b) Portography acquired immediately before portal vein embolization shows a normal portal vein anatomy. Green arrow: right portal vein; red arrow: left portal vein. (c) Portography immediately after portal vein embolization shows satisfactory occlusion of the anterior and posterior sectorial portal vein branches. Red arrow: left portal vein; green arrows: right portal branches occluded. (d) Computed tomography 4 weeks after portal vein embolization shows a significant increase in left liver volume (hypertrophy rate of 51%). Red arrows: coils placed in the right portal vein branches; black arrows: definition of the liver ischemic line between the right and left hepatic lobes.

Since FLR volume correlates with the development of PLF, a systematic assessment of liver volumetry during preoperative planning is critical, especially in the setting of baseline liver dysfunction or anticipated extended hepatectomy [16]. Hepatic contrast-enhanced CT, with a 5.0 mm or less slice thickness, with a 16-detector row multislice CT scanner (Siemens) was performed prior to and 4–7 weeks after PVE. On single slices, the both total liver, tumor, and FLR (accordingly to previously surgical planning) were delineated with a handheld cursor using a freely downloadable open-source image analysis software package: OsiriX®—a validated software for liver volumetric evaluation [17]. When the total regions of interest were selected within one series, the volumetric calculations were obtained using OsiriX® by multiplying surface and slice thickness and then adding up individual slice volumes [17]. TFLV was defined as the total hepatic volume subtracted by the tumor volume. FLR was defined as the portion of the liver that would persist after liver resection. The ratio between the FLR and the TFLV was calculated and defined as the FLR/TFLV ratio. The increase in the FLR after PVE was also quantified and calculated by the formula (FLR after PVE−FLR before PVE) ÷ (FLR before PVE) as suggested in guidelines [14]. (Figures 2(a)–2(d))

### 2.6. Secondary Outcome Evaluations

For all 64 patients incorporated in our study, clinical, imaging, and laboratory data were scrutinized to the most updated available information up to July 2017. Liver function tests, including serum levels of total bilirubin (TB), aspartate aminotransferase (AST), and international normalized ratio (INR) were measured prior to PVE and surgery. Patients were analyzed for tumor type, administration of systemic chemotherapy before PVE, number of chemotherapy cycles, type of systemic chemotherapy administered, number of PVA vials and coils per patient in each PVE procedure, major and minor adverse events after PVE, submission to the planned liver surgery, reasons for not performing the previously deliberate surgery, surgical complications, period of hospitalization, and death after PVE and surgery. Adverse events were categorized as proposed in previous publications [18, 19] and considered major if they triggered (>48 h) or prolonged hospitalization and required unintentional increment in level of care or resulted in long-lasting adverse effects and death [20]. Minor complications were categorized as those which required minimal therapy or prolonged hospitalization for observation only [21]. Survival was calculated to compare patients submitted or not to the planned hepatic surgery after PVE.

### 2.7. Statistical Analysis

Mean, standard deviation, and range were estimated for numerical variables as descriptive statistics, while absolute numbers and percentages were calculated for categorical variables. Paired *t*-test or paired Wilcoxon rank-sum test, as appropriated, were used to compare TFLV and FLR volumes before and after PVE. To test associations between liver volumes before and after PVE (e.g., FLR/TFLV ratio before and after PVE), linear regression models were used. The association between variables (e.g., liver tumor histology and FLR increase) was tested using Fisher's exact test and chi-squared test. A *p* value below 0.05 was considered significant. All statistical analyses were performed using R software. The confidence intervals are based on a 95% confidence level. Survival rates were calculated from the date of PVE with Kaplan–Meier methods.

## 3. Results

The baseline clinical characteristics of the 64 patients are summarized in Table 1.

There were 45 (70%) men and 19 (30%) women with a mean age of 64 years ± 12 (range, 42–84 years). Of these 64 patients, 47 (73%) patients were diagnosed with colorectal liver metastases, 12 (19%) patients with cholangiocarcinoma, 4 (6%) patients with hepatocellular carcinoma, and one (2%) patient with hydatid cyst. Liver cirrhosis was diagnosed in two (3%) patients. Forty-one (64%) patients were submitted to systemic chemotherapy before PVE, and the most frequent type of systemic chemotherapy was FOLFIRI (*n* = 9.23%).

PVE was performed successfully in all 64 patients. Embolization required a mean number of 7.75 ± 2.9 vials of PVA and 9.73 ± 4.2 coils. No coil migration was reported on the cohort. One patient had biliary obstruction at presentation and was percutaneously drained previously PVE. In 63 (98%) patients, the ipsilateral approach was adopted in contrast with 1 patient, in which the contralateral option was required for PVE due to large tumor volume precluding safe access through the right liver lobe. Mean hospital stay was 2.6 days ± 1.61 after PVE. Fifty-three (83%) patients had a right PVE, two (3%) patients had a right PVE plus segment IV (RPVE + IV) embolization, one (1%) patient had a right PVE plus right hepatic vein embolization, five (8%) patients had a left PVE, and three (5%) patients had a left PVE plus right anterior sectorial embolization.

### 3.1. Volumetric Liver Results and Laboratory Values

**Figure 3:**
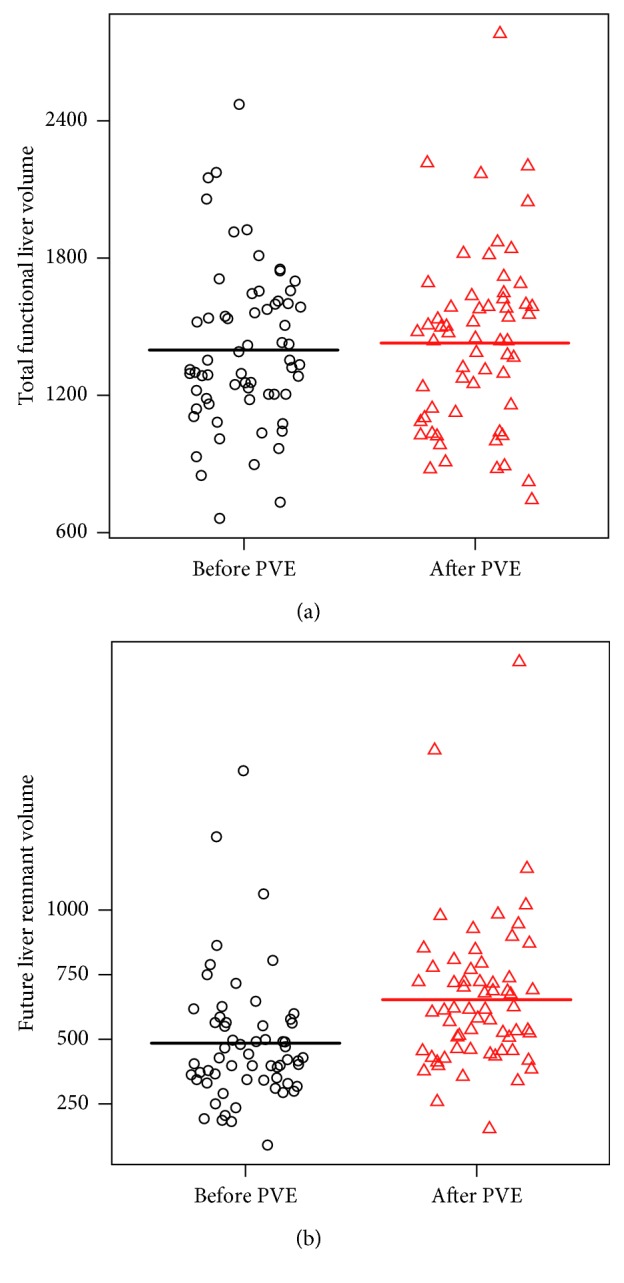
(a) Total functional liver volume before and after portal vein embolization (in milliliters). Differences were not statistically significant. (b) Future liver remnant volume before and after portal vein embolization (in milliliters). Differences were statistically significant (*p* < 0.001).

After PVE patients were submitted to volumetric CT to assess FLR growth with a median time interval of 36.2 ± 14.4 days. FLR increased from a mean value of 484 ml ± 242 to 654 ml ± 287 (*p* < 0.001) after PVE, corresponding to a mean FLR increase of 40% ± 29% and a mean FLR/TFLV ratio increase of 11% ± 5%. The TFLV increased from 1399 ± 347 to 1428 ± 380 after PVE (Figure 3).

Tumor volume increased from a mean value of 114 ml ± 377 to 138 ml ± 386 after PVE. Right liver volume decreased from a mean value of 985 ml ± 393 to 853 ml ± 386 after PVE (Table 2).

**Figure 4:**
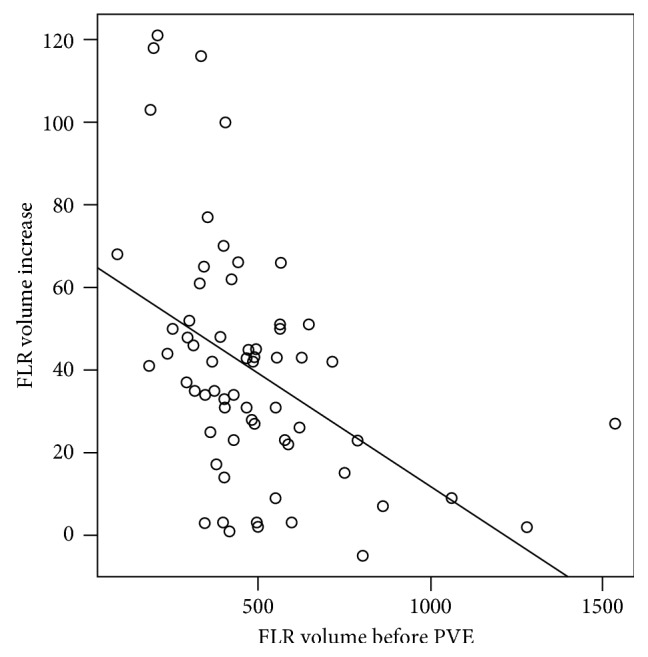
Future liver remnant volume increase versus future liver remnant volume before PVE. There was a negative correlation between those two variables, demonstrating that those patients with the smallest FLR volumes obtained superior volume increase after PVE.

Laboratory data, regarding total bilirubin, AST, and INR before PVE and before surgery, were 1.41 ± 2.37 and 2.08 ± 5.24; 40 ± 23.63 and 55.94 ± 76; 1.07 ± 0.15 and 1.22 ± 0.45, respectively. There was an inverse (negative) relation between the FLR volume before PVE and FLR volume increase induced by PVE (correlation coefficient = −0.46; *p* < 0.001) (Figure 4).

### 3.2. PVE Adverse Events

Two out of 64 patients submitted to PVE experienced major adverse event (3.1%): 1 case of left hepatic artery branch laceration and 1 case of hemoperitoneum and hemothorax. The first patient was a 73-year-old man with colorectal liver metastases, submitted to right PVE, through a contralateral puncture, due to extensive metastatic burden in the right liver lobe. During the procedure, unintended left hepatic artery branch laceration occurred, with immediate perihepatic hematoma formation. A femoral arterial access was established but no evidence of active bleeding was seen on dedicated angiography, suggesting interruption of the arterial bleeding. The patient remained stable and was discharged 4 days later. The latter patient was a 71-year-old female with cholangiocarcinoma. Two hours after PVE, the patient developed signs of hemorrhagic shock, and a hemoperitoneum and hemothorax were diagnosed. An angiography was performed, and no active bleeding was depicted. There was no need for thoracic drainage. No underlying etiology was found, and this patient also recovered well. This event prolonged her hospital stay for 6 days. Four patients had minor complications (6.2%) with 3 cases of fever and 1 case of nausea and vomiting (Table 2).

### 3.3. Surgical Outcomes

Twenty patients (31.2%) were not submitted to surgery as a result of disease progression (*n* = 17), liver insufficiency (*n* = 1), insufficient FLR volume, and disease progression (*n* = 1), and one patient declined surgery (*n* = 1). A total of 44 (68.8%) patients underwent liver surgery, and the performed hepatic procedures are listed in Table 3.

**Figure 5:**
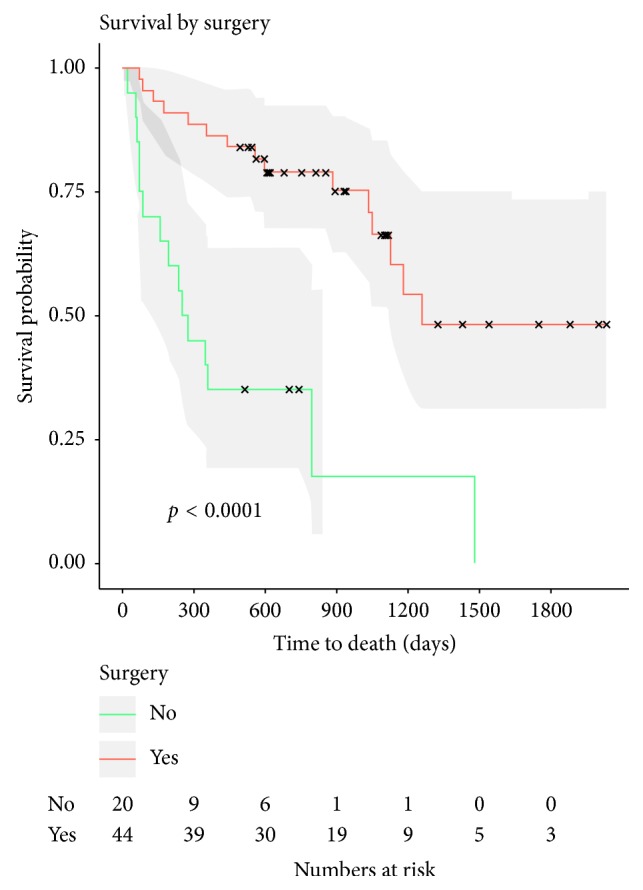
Overall survival according to surgery. Accomplishment of the planned liver surgery was associated with better overall survival when compared to those patients in whom surgery was declined (*p* < 0.001).

Complications during and immediately after hepatic resection were (Table 4) biliary fistula (*n* = 1), intraoperative hepatic bleeding (*n* = 1), abscess (*n* = 2), principal biliary duct laceration (*n* = 1), and portal vein and small bowel laceration (*n* = 1) that were successfully managed. Postoperative hepatic insufficiency was reported in one patient who died 32 days after surgery. Surgical-related mortality was thus 2.3% (*n* = 1). Mean hospital stay was 18 days ± 14.58 after liver surgery. Accomplishment of the planned liver surgery was related with better overall survival in contrast with those patients in whom surgery was declined (*p* < 0.001) (Figure 5).

The preoperative data of the patients are listed in Table 3.

## 4. Discussion

Currently, preoperative PVE is an important technique to be considered, in the proper clinical setting, before major hepatectomy. This procedure helps diminish postoperative morbidity and mortality through the achievement of a sufficient nontumoral liver—FLR—volume precluding the occurrence of postoperative liver failure that may be present in up to 20% of patients [5]. In the present cohort liver, failure after PVE and surgery was reported in only 1 patient (2.3%) highlighting the importance of presurgical PVE.

One of the fundamental aspects of PVE is the elected embolic material. The best agent is one which originates permanent embolization without recanalization, has a significant toleration by the patient, and is effortless to administer [13]. PVA particles are secure, cause minor periportal reaction, and originate long-lasting portal vein occlusion when they are used in combination with coils [22]. Nevertheless, a recent systematic review [11] and two retrospective studies [23, 24] reported that PVE with N-butyl-cyanoacrylate (NBCA) had a more robust effect in FLR hypertrophy than PVE with PVA and coils. Moreover, a study performed by de Baere et al. [25], in an animal model, showed that PVE with NBCA induced a significantly greater increase in hepatic lobules volume when compared with other embolic materials. Although there seems to be a significant benefit in FLR hypertrophy with the use of NBCA, no prospective randomized trials approaching this topic are currently available. This adhesive embolic material, NBCA, that might be more efficient, does require specific and dedicated training and is associated with nontarget embolization [26]. In addition, considering the use of vascular plugs in PVE, according to one study [27], there were no significant differences between PVA plus coils, PVA plus plug, and PVA plus plug and coils regarding future liver remnant hypertrophy after PVE.

Since one of the most important properties of an embolic material is its capacity to induce liver growth when used for PVE, we wanted to evaluate this specific outcome in our own series of patients. In our retrospective cohort of 64 patients submitted to PVE with PVA plus coils, we obtained a 40% increase in the FLR after a median of 36 days. Compared with other published hypertrophy rates, our results were equal or superior to those of most previous studies [13, 27, 28]. Some series showed a higher hypertrophy response, as in the Kishi et al. study [29]. However, it is difficult to establish a direct comparison of our results to the latter and other studies due to relevant technical differences such as segment IV portal vein embolization. In their study, Kishi et al. reported a high FLR hypertrophy rate of 54% after RPVE + IV embolization. Interestingly, in the study by Madoff et al. [13], it was also demonstrated a higher hypertrophy rate after right PVE + IV embolization, even though that higher value was similar to that reported in our own study without segment IV embolization. Furthermore, the real benefit of segment IV portal vein embolization is still not clear. A study by de Baere et al. reported an increase in the FLR of 68% and 69% after right PVE and RPVE + VI embolization, respectively, showing no difference in hypertrophy rates when segment IV embolization was performed [30]. This study also demonstrated a superior FLR rate compared with our results, although differences among these studies make it difficult to establish linear comparison.

One relevant aspect of our study was the adoption of PVA as the embolic material. Madoff et al. [31] showed that tris-acryl microspheres performed favorably, in hypertrophy results, than PVA for PVE plus segment IV embolization. While these results do suggest that it might be possible to obtain better regenerative results with tris-acryl microspheres, they had a different study population (they only included patients submitted to right PVE + IV), and using numerous tris-acryl microspheres vials would drastically increase the cost of PVE at our institution since it is significantly more expensive in our local setting.

Two major complications after PVE were recorded in our series (3.1%), consisting of one case of hemoperitoneum and hemothorax and one case of left hepatic artery laceration. The latter patient was a 73-year-old man with colorectal liver metastases, underwent right PVE through a contralateral approach, and was the only patient in our study on whom the FLR puncture was performed. This major complication might be explained by the use of the contralateral approach. The contralateral approach has known advantages such as direct catheterization of the desired portal branches, use of shorter catheters, and avoidance of tumor transgression in high tumor burden patients. Nevertheless, the contralateral puncture is somewhat trickier in patients with very small FLRs and disadvantageous body habitus and has the inherent disadvantage of risking injury to the FLR [32]. However, the largest study, comprising 188 patients, concerning PVE complications suggested that the contralateral approach does not impose higher risks compared with the other performed approaches [18].

In our study, 31% of the patients were not submitted to hepatic surgery, which is slightly more than the reported in a recent systematic review [11] where 20% (358/1, 791) of the initially planned hepatectomies after PVE were cancelled. Most of these patients had tumor progression. We only found 16% of complications after liver surgery, which is considerably below the complication rates (25%–30%) reported in most similar series [30, 33, 34]. This might reflect a more rigorous criterion for surgery selection and a higher cancelation rate of the originally planned liver resection [13, 35].

Our study has limitations, such as the retrospective design and the exclusion of part of the cohort from the analysis due to missing imaging data. The strengths of our study were the application of the same PVE technique along many years of practice and the overall homogeneous patient population comprised almost exclusively by noncirrhotic patients, which would otherwise have puzzled our hypertrophy outcomes due to the known effects of cirrhosis in liver regeneration [36].

In conclusion, we demonstrated herein that PVE with PVA and coils could be accomplished with a low incidence of major complications. It is also associated with a high FLR hypertrophy yield and enables patients to be submitted to the potentially curative treatment of liver tumor resection with minimal postoperative liver failure rates.

## Figures and Tables

**Table 1:** Patients' characteristics.

Number of patients	64
Age, mean (SD)	63.84 (11.56)
Sex, *N* (%)	
Female	19 (29.69)
Male	45 (70.31)
Tumor type, *N* (%)	
Hepatocellular carcinoma	4 (6.25)
Colangiocarcinoma	12 (18.75)
Colorectal metastases	47 (73.44)
Hydatid cyst	1 (1.56)
Cirrhosis, *N* (%)	
Absent	62 (96.88)
Present	2 (3.12)
Cirrhosis etiology, *N* (%)	
HCV	1 (1.56)
None identified	63 (98.44)
Chemo before PVE, *N* (%)	
No	23 (35.94)
Yes	41 (64.06)
Type of systemic chemotherapy, *N* (%)	
FOLFIRI	9 (23.08)
FOLFIRI + bevacizumab	2 (5.13)
FOLFIRI + cetuximab	5 (12.82)
FOLFIRI + panitumumab	1 (2.56)
FOLFIRINOX	1 (2.56)
FOLFOX	6 (15.38)
FOLFOX + bevacisumab	2 (5.13)
FOLFOX + cetuximab	2 (5.13)
FOLFOX + folfirinox	1 (2.56)
FOLFOX + folfirinox + cetuximab	1 (2.56)
XELOX + cetuximab	1 (2.56)
XELIRI	1 (2.56)
Xeloda + FOLFIRI + erbitux	1 (2.56)
XELOX	3 (7.69)
XELOX + bevacizumab	2 (5.13)
XELOX + XELIRI	1 (2.56)
Chemo cycles, mean (SD)	3.38 (4.36)
Biliary drainage before PVE, *N* (%)	
No	63 (98.44)
Yes	1 (1.56)
Arterial embolization, *N* (%)	
No	64 (100)

HCV: hepatitis C virus; PVE: portal vein embolization.

**Table 2:** PVE and main outcome.

Number of patients	64
PVE segments, *N* (%)	
Right	53 (82.81)
Right + IV	2 (3.12)
Right + RHV	1 (1.56)
Left	5 (7.81)
Left + ARS	3 (4.69)
PVE ipsi or contralateral, *N* (%)	
Contra	1 (1.56)
Ipsi	63 (98.44)
PVA total vials, mean (SD)	7.75 (2.93)
Total coils, mean (SD)	9.73 (4.21)
Adverse events, *N* (%)	
Fever	3 (4.69)
Hemoperitoneum and hemothorax: angiography did not reveal active bleeding	1 (1.56)
Left arterial branch lateration	1 (1.56)
Nausea and vomiting	1 (1.56)
None	58 (90.62)
Hospital stay in days, mean (SD)	2.59 (1.61)
TFLV, mean (SD)	1399.02 (346.92)
TFLV after PVE, mean (SD)	1428.62 (379.58)
FLRV, mean (SD)	484.31 (241.64)
FLRV after PVE, mean (SD)	653.61 (286.66)
Right liver volume before PVE, mean (SD)	984.89 (393.31)
Right liver volume after PVE, mean(SD)	853.06 (386.42)
Tumor volume before PVE, mean (SD)	114.03 (377.4)
Tumor volume after PVE, mean (SD)	137.76 (385.8)
Increase in the FLR ratio, mean (SD)	11.14 (4.83)
Increase in the FLR percent degree of hypertrophy, mean (SD)	40.16 (28.75)

PVE: portal vein embolization; RHV: right hepatic vein; ARS: anterior right sector; TFLV: total functional liver volume; FLRV: future liver remnant volume; FLR: future liver remnant.

**Table 3:** Patient outcome.

Total of patients	64
Type of hepatectomy, *N* (%)	
RH	21 (47.73)
RH + I	4 (9.09)
RH + I + IV	1 (2.27)
RH + IV	10 (22.73)
LH	6 (13.64)
LH + V/VII	1 (2.27)
Tx	1 (2.27)
Reason for no surgery, *N* (%)	
Liver failure	1 (5.00)
Insufficient volume + disease progression	1 (5.00)
Disease progression	17 (85.00)
Patient declined surgery	1 (5.00)
Total bilirubin before PVE, mean (SD)	1.41 (2.37)
Total bilirubin before surgery, mean (SD)	2.08 (5.24)
AST before PVE, mean (SD)	40.41 (23.63)
AST before surgery, mean (SD)	59.94 (76)
INR before pve, mean (SD)	1.07 (0.15)
INR before surgery, mean (SD)	1.22 (0.45)

RH: right hepatectomy; LH: left hepatectomy; Tx: transplant; PVE: portal vein embolization; AST: aspartate aminotransferase; INR: international normalized ratio.

**Table 4:** Patient outcome: surgical complications.

Surgical complications, *N* (%)	
Principal biliary duct laceration	1 (2.13)
Abscess	2 (4.26)
Biliary fistula	1 (2.13)
Hemorrhage	2 (4.26)
Hepatic failure	1 (2.13)
Portal vein and small bowel laceration	1 (2.13)
None	39 (82.98)
Length of hospital stay, mean (SD)	17.72 (14.58)

## Data Availability

The patient data (baseline patient clinical characteristics, imaging records, liver volumetric data, and postoperative course) used to support the findings of this study are available from the corresponding author upon request.

## Abbreviations

PVE: Portal vein embolization
FLR: Future liver remnant
PLF: Postoperative liver failure
TFLV: Total functional liver volume
CT: Computed tomography
MR: Magnetic resonance
PVA: Polyvinyl alcohol
TB: Total bilirubin
AST: Aspartate aminotransferase
INR: Index normalized ratio
NBCA: N-butyl-cyanoacrylate
RPVE: Right portal vein embolization.
